# FINDEXER: a new technique to find unit cells in sparse serial patterns

**DOI:** 10.1063/4.0001055

**Published:** 2025-10-27

**Authors:** Daniel Paley, Aaron Brewster, David Mittan-Moreau, Nicholas Sauter

**Affiliations:** Lawrence Berkeley National Laboratory, Berkeley, CA, USA

## Abstract

XFEL microcrystal diffraction has been an ongoing success for chemical crystallography, with dozens of structures determined since 2022. For unknown samples, the most challenging step is finding the unit cell. In typical experiments, we have failed to determine the unit cell for about 50% of our samples, which means that the unit cell problem presents an opportunity to double our success rate. In this talk I will present a new algorithm that uses 3-dimensional spot pair relationships to determine unit cells with high accuracy and reliability.

The FINDEXER algorithm uses spot pairs, with two lengths and one angle (q1, q2, theta), as the fundamental input data. We harvest ∼hundreds of frequently observed spot pairs from a serial diffraction dataset. Then we build up the 6 unit cell parameters in a stepwise manner, using an intermediate 2d sub-basis that indexes a fraction of the data. (For example, a sub-basis constructed from the (100, 001) spot pair would index all spot pairs that have both k-indices equal to 0.) The full 3d basis is completed by analyzing spot pairs that are half-indexed in the sub-basis. Finally, we test cell-doubling transformations to account for possible space-group extinctions in the initial sub-basis.

We believe this is the first algorithm that uses 3d spot relationships in non-oriented, X-ray-wavelength, sparse patterns for unit cell determination. Indexing is rapid and computationally lightweight. I will give a full outline of the FINDEXER algorithm and a short selection of new structures enabled by the method.

